# Is it Effective and Cost-saving to Send all Tonsillectomy Specimens for Histopathological Examinations?

**DOI:** 10.22038/IJORL.2021.57235.2976

**Published:** 2022-01

**Authors:** Mohammad Faramarzi, Mohsen Ghaffari Darab, Abdosaleh Jafari, Hatam Salehpour, Masoud Janipour, Sareh Roosta, Khosro Keshavarz

**Affiliations:** 1 *Otolaryngology Research Centre, Department of Otolaryngology, School of Medicine, Shiraz University of Medical Sciences, Shiraz, Iran.*; 2 Joint* doctoral student with Deakin University (Australia) and University of Bayreuth (Germany), Researcher at Institute for Medical Management and Health Sciences (IMG)*; 3 *Health Human Resources Research Centre, School of Health Management and Information Sciences, Shiraz University of Medical Sciences, Shiraz, Iran. *; 4 *MSc in Biostatistics, MA in General Psychology, Otolaryngology Research Centre, Shiraz University of Medical Sciences, Shiraz, Iran.*; 5 *Emergency Medicine Research Center, Shiraz University of Medical Sciences, Shiraz, Iran.*

**Keywords:** Aspirin Intolerance, Asthma, Desensitization, Endoscopic Sinus Surgery, Nasal Polyps, Sinusitis

## Abstract

**Introduction:**

The present study aimed to investigate the necessity or unnecessity of sending all tonsillectomy specimens for pathological examinations in Shiraz, Iran; moreover, it examined malignancies, cost-saving, causes, and the ways to prevent sending all specimens.

**Materials and Methods:**

In the first retrospective phase of the study, a checklist was used to gather demographic, clinical, and cost information of 18,437 tonsillectomy specimens during 2004-2018 in Shiraz, Iran. In order to estimate the cost of each pathology specimen, the cost components, including human resources costs and consumables, were collected in the private and public sectors separately and divided by the number of cases performed. Finally, the financial burden resulting from these services (in the study centers) was calculated by multiplying each item's cost by the total number of these services.

**Results:**

Out of the total 18,437 histopathology specimens examined, only 118 (0.64%) samples were identified with unusual diagnoses, of which 66 (56%) cases had malignant tumors, and the remaining 52 (44%) samples included benign tumors (n=41), infections (n=2), and other problems (n=9). The second phase results also indicated that according to the ear, nose, and throat experts, the rules and regulations governing the country's health system and the suspicion of tumors were the main reasons for sending tonsil specimens for pathological examinations. Generally, the annual cost-saving rates in the studied public and private centers were $87,919 and $179,530 purchasing power parity, respectively.

**Conclusions:**

According to the results, sending tonsillectomy specimens should only be limited to nonroutine ones for economic-clinical reasons.

## Introduction

Tonsillectomy is one of the most common ear, nose, and throat (ENT) surgeries globally, used in an extensive range of indications in adults and children, many of which are performed for benign or non-neoplastic procedures (1). Moreover, upper airway obstruction and recurrent tonsillitis are considered the most common indications (2).

Current medical protocols make it legally mandatory to perform a pathological examination of any surgical specimens; however, there are pieces of evidence that have questioned the necessity of this approach in terms of health economics. Pathological examinations for any specimen have costs that will not only be imposed on the patients and families in the current state of the health care system but in part on the relevant organization as well. The fact is that tonsillar malignancies are rare. Indeed, doing merely standard histopathological and microscopic studies on all tonsillectomy specimens without providing a proper clinical index may be a dispensable expense that will result in using a significant part of health care resources (3). In the United States, about one million tonsillectomy specimens are annually sent to pathology laboratories, the examination of each may cost $69. Therefore, $68,000,000 is annually spent on tonsillectomy examinations in the United States, while the prevalence of malignancy in all age groups is only about 0.015% (4,5). Other studies also indicated that malignancy in pathological specimens was negligible. Yonnis et al. studied 2,438 pathological specimens and reported no unexpected malignancy, and the pathological and microscopic examinations cost approximately $5 and $13, respectively; therefore, a total of $597,310 was saved on the pathological reports in their study (3).

The main reason for the investigation of the need for pathological examination of routine tonsillectomy specimens is to reduce costs and time spent by the workforce, which is a significant issue nowadays. On the other hand, this approach should not lead to unpredictable diseases, especially malignancies, to be missed, and have an adverse effect on disease prognoses by delaying identifying required treatments (6).

The present study dealt with pathological diagnoses of all tonsillectomy specimens during 2014-2018, diagnoses of malignancies in adults and children, and the costs of pathological evaluations in two university referral hospitals in Shiraz, Iran. In addition, reviewing credible scientific sources and official reports would lead to proposing a cost-effective and reasonable approach for decision-making on saving time and costs, and only the tonsillectomy specimens requiring pathological examinations would be selected and referred to laboratories.

Since the regulations governing medical centers in Iran have made it necessary to send all tonsillectomy specimens for pathological examinations, this study aimed to identify and investigate malignancies and cost-savings by not sending routine tonsillectomy specimens for histopathological examinations.

## Materials and Methods

This applied and analytical-descriptive study was carried out in two phases, as described in the following.

Phase One

In the first phase, the information of 18,437 tonsillectomy specimens sent during 2004-2018 to the pathology departments of Khalili and Shahid Dastgheib educational hospitals affiliated to Shiraz University of Medical Sciences, Shiraz, Iran, were retrospectively collected and examined to decide on the necessity or unnecessity of sending them. No sampling was performed at this stage, and analysis was made on all pathological reports related to the tonsillectomy specimens sent to the pathology departments of the mentioned medical centers within 15 years (2004-2018). 

The information of all pathological documents related to the tonsillectomy specimens sent to pathology laboratories of Khalili and Shahid Dastgheib Hospitals within the 15 years were examined by age and gender. The reports of different pathologies were listed in the order of prevalence, and the pathologies unexpected to the surgeons were set apart, and their percentage was determined and compared with other published studies. Moreover, the costs of performing tonsillectomy specimen pathologies in public and private sectors were estimated separately. The cost of each specimen pathology in the public sector was calculated. All the costs of the pathology laboratories in the medical centers under study were collected separately (e.g., salaries, equipment, building depreciation, consumables, energy consumption costs).

Similarly, the costs of the private sector were extracted in several private referral centers, and the authors finally calculated the cost of performing each specimen pathology in public and private sectors by attributing all the costs and summing them up, and then dividing the total costs by the total numbers of the pathological specimen. Ultimately, the financial burden of examinations was calculated by multiplying each specimen's cost by the total annual cases in the studied centers. The costs were calculated based on 2018 tariffs, and the international comparison based on the purchasing power parity (PPP) was performed with the exchange rate of each dollar equal to 12,800 Rials (7).

Furthermore, a literature review was conducted, and the experts' opinions related to the research objectives and the current situation were asked. Afterward, a researcher-made checklist, which included demographic, clinical, and cost information, was used in the first phase to collect the required data. In order to collect the data on the effectiveness of the current method (sending all specimens to pathology departments), the results of international studies were used, and it was investigated whether to send tonsillectomy specimens or not. The cost-effectiveness information obtained from the previous stage was applied to investigate the economic and clinical dimensions of performing pathological examinations, compared to the time when these examinations are not carried out. As mentioned above, reviewing the pathological reports and calculating the costs based on financial documents resulted in estimating each specimen's mean cost in the public and private sectors. Furthermore, the ratio of malignancies to the total specimens was identified by age and gender. In addition, the Excel software was used to calculate the costs and determine the ratio of malignancies.

Phase Two

In the second phase, a researcher-made questionnaire on the causes and strategies for the prevention of sending tonsillectomy specimens for pathological tests was used to examine the experts' opinions. The questionnaire consisted of 10 questions, 6 of which addressed the causes of sending tonsillectomy specimens, and 4 were on the prevention of sending tonsillectomy specimens. The reliability of the questionnaire was obtained at 0.87 in a preliminary study using Cronbach's alpha method. At this stage, the statistical population comprised all the 187 ENT specialists throughout the country; moreover, the sample size was determined to be 126 using the Krejcie and Morgan table (8), and the subjects were selected through a simple random sampling method. Their attitudes were then asked through an email or a phone call, and 48 fully-completed questionnaires were finally received. The data were analyzed in SPSS software (version 22) through the chi-square statistical test. The results of this research can be used at decision-making levels in the Ministry of Health and insurance organizations to decide on sending or not sending tonsillectomy specimens for pathological examinations.

## Results

The findings of the first phase of the study are presented in two stages, as described in the following. In the first stage, the specifications and results of the histopathological examinations of all tonsillectomy specimens during the last 15 years were provided. The second stage dealt with the results of estimating the total direct costs of the pathology of each tonsillectomy specimen, and finally, the results of the chi-square test were presented in the second phase.

***Phase One***

***First-Stage Findings***

The present study investigated the total number of tonsillectomy specimens (n=18,437) sent to the pathology departments of Khalili and Dastgheib Hospitals for histopathological examinations during 2004-2018. Among all the patients in the age range of 10 months to 92 years, the majority (n=9,403; 51%) of the cases were male, and the mean age was obtained at 13.65 years. Moreover, 13,588 (73.7%) subjects were <18 years old. As indicated in Table 1, only 118 out of the total 18,437 specimens that underwent histopathological examinations were diagnosed abnormal or suspected. Only 66 out of these 118 specimens had malignant tumors, and among the remaining (n=52), 41, 2, and 9 specimens had benign tumors, infections, and other problems, respectively. An average of 7.9 abnormal cases was diagnosed annually, approximately 4.4 of which had malignancies. Moreover, as shown in Table 1, 105 (89%) out of 118 abnormal cases were over 18 years old. It is noteworthy that among 66 malignancies, only 4 (6%) cases were under 18 years, and the remaining 62 (94%) were over 18. In addition, 13%, 15%, 72%, 52%, 20%, and 28% of the malignant cases had tonsil ulcer, lymphadenopathy, tonsil enlargement, pain dysphagia tonsil ulcer, voice change snoring, and neck masses, respectively. The results also revealed that physicians were suspicious of malignancies in 66 malignant cases before an operation.

**Table 1:** Characteristics of unusual cases detected by histopathological tests among all examined cases of tonsillectomy specimens during 2004-2019

Diagnosis	≤18yr	<18yr	Total	Case	≤18yr	>18yr	Total	Unexpected Abnormalities
Malignant neoplasm	4	62	66					
				Non-Hodgkin's L.	-	39	39	One case of non-Hodgkin's L.
				Hodgkin's L.	-	2	2	
				Burkitt's L.	4	-	4	
				Mucoepidermoid carcinoma	-	1	1	
				Squamous cell carcinoma	-	9	9	
				Nasopharyngeal carcinoma	-	2	2	
				Poorly differentiated carcinoma	-	1	1	
				Synovial sarcoma	-	5	5	
				Diffuse large B-cell lymphoma	-	3	3	
*Benign neoplasm and non-neoplastic masses*	9	32	41					
				Squamous papilloma	1	-	1	One case of Lymphoid polyp and one case of Lymphoepithelial cyst
				Lymphoid polyp	3	-	3	
				Fibroepithelial polyp	-	7	7	
				Lymphoepithelial cyst	-	1	1	
				Epithelial inclusion cyst	4	19	23	
				Transformed germinal center	-	1	1	
				Severe inflammation	1	-	1	
				Granulomatous inflammation	-	1	1	
				Pleomorphic adenoma	-	3	3	
*Infectious processes*	-	2	2					
				TB	-	1	1	
				Abscess	-	1	1	
*Other pathologic findings*	-	9	9					All eight cases of epithelial inclusion cyst
				Calcification and stone	-	2	2	
				Cartilaginous metaplasia	-	3	3	
				Foreign bodies	-	3	3	
				Branchial fistula	-	1	1	

Second Stage Findings

The information obtained by estimating total direct costs of the histopathology of each tonsillectomy specimen was used, and the results were presented based on the cost of the histopathology of each specimen in the public and private sectors separately. The financial burden was estimated by examining the average number of samples sent in one year to two state centers (n=1,229) and all state centers (n=3,047) in Fars province, Iran, for pathology tests. It should be noted that due to the lack of access to all tonsillectomy operations in the country, the average number of tonsillectomy operations in one year could only be obtained from Shiraz University of Medical Sciences, Shiraz, Iran, and its financial burden was estimated. Therefore, a total of 3,047 tonsillectomies were performed in all state centers of the province in one year. Table 2 indicates the total direct cost of histopathology of each tonsillectomy specimen, including personnel expenses, equipment depreciation, consumables, and the energy consumed for all pathology specimens per year. Furthermore, the costs of each tonsillectomy specimen histopathology in public and private sectors were estimated separately. As demonstrated in the results, the mean direct costs of performing histopathology of each tonsillectomy specimen in public and private sectors were $72 and $147 PPP, respectively. Moreover, 63%, 18%, 4%, and 6% in the public sector and 73%, 7%, 12%, and 2% in the private sector were allocated to the costs of the workforce, equipment depreciation, consumables, and energy consumption, respectively. In addition, the total direct cost of performing 1,229 and 3,047 histopathology tests in one year in the public sector was estimated at $88,488 and $219,384 PPP, respectively. However, the first phase of the study indicated that an average of 7.9 abnormal cases was diagnosed per year, the cost of which was $569 PPP.

**Table 2:** Estimation of the total of direct costs spent on histopathological examination of each tonsillectomy specimen

Costs Items	Mean Costs per Specimen (PPP$)				Total Costs for All the Specimens Per Year (PPP$)	
	**Public sector**	**Percentage**	**Private sector**	**Percentage**	Study centers**	All public centers***
Personnel*	45	63%	108	73%	55,305	137,115
Depreciation of equipment	13	18%	11	7%	15,977	39,611
Depreciation of building	7	10%	7	5%	8,603	21,329
Materials and supplies (color, paraffin, tissue fluids)	3	4%	18	12%	3,687	9,141
Municipal services (water, electricity, gas, and telephone)	4	6%	4	2%	4,916	12,188
Total Costs	72	100%	147	100%	88,488	219,384

*Personnel: Physician, specialist technician, receptionists, and clerks, **Based on 1,229 cases per year on average at our institution. ***Based on 3,047 cases per year on average at our public centers in the province

**Estimation of Cost-Saving Due to Not Sending Routine Tonsillectomy Specimens for Histopathology**

The results indicated that sending all specimens for pathological examinations was not necessary, and only if the patient had other clinical symptoms indicating malignancy, the specimen was needed to be pathologically examined. According to the results, an average of 1,229 tonsillectomy specimens was annually examined in the studied medical centers, only about 7.9 cases of which were necessary, and the remaining (n=1,221.1) were routine tonsillectomy specimens. 

Therefore, not sending routine cases for pathological examinations would save approximately $87,919 per year only in the two public health centers under investigation. The cost-saving rate would be higher in private centers because the cost of each specimen pathology is currently about $147 PPP and higher than that in public centers; therefore, by not sending 1,221.1 specimens, the cost-saving could be around $179,530.

**Phase Two**

**Statistical Analysis**

The chi-square statistical test was used to investigate the data obtained from the research and answer the questions. The results of data analysis are presented in the following.

**Causes of Sending the Specimens for Pathology Tests**

As indicated in Table 3, a total of 48 ENT specialists believed that the rules and regulations governing the country's health system, suspected masses, doubts about diagnoses of uncommon pathogens, and obtaining pathologist approvals to complete the final process had a significant effect on sending tonsillectomy specimens for pathology (P<0.01). Therefore, it could be stated that with a 99% confidence interval, the factors mentioned above were the main reasons for sending tonsillectomy specimens for pathology tests. 

However, other factors, such as patient insistence and being routine (normality), had no significant effect on sending tonsillectomy specimens for pathology tests (P>0.01).

**Table 3:** Results of statistical data analysis (Subjects' attitudes towards the causes of sending tonsil samples for pathology)

**P-value**	**Chi-square**	**Subjects' Attitudes**					**Statistical indices of variables**
		**Completely Disagree**	**Disagree**	**No Idea**	**Agree**	**Completely Agree**	
0.001	19.29	2	4	9	16	17	Laws and regulations governing the country's health system
0.001	70.17	0	2	3	6	37	Doubts about tumors
0.008	13.87	3	6	9	18	12	Doubt about diagnosing uncommon pathogens
0.255	5.33	10	14	6	12	6	Patient insistence
0.001	20.96	5	8	8	22	8	Obtaining the pathologist's approval to complete the final procedure
0.551	3.04	9	10	14	7	8	Being routine (common)
							

**Prevention Strategies**

As shown in Table 4, obtaining complete records of patients, conducting thorough examinations on patients, modifying the rules and regulations governing the country's health system, establishing think tanks, and providing guidelines, are the strategies to prevent sending tonsillectomy specimens for pathological examinations (P<0.01). Accordingly, it could be stated that with a 99% confidence interval, these factors might help prevent the sending of tonsillectomy specimens for pathological tests.

**Table 4:** Results of statistical analysis of data (Subjects' attitudes towards the strategies for the prevention of sending tonsil specimens for pathology)

**P-value**	**Chi-square**	**Subjects' Attitudes**					**Statistical indices of variables**
		**Totally Disagree**	**Disagree**	**No Idea**	**Agree**	**Totally Agree**	
0.001	21.67	0	9	1	15	23	Getting a complete history of the patient
0.001	34.17	0	3	1	20	24	Conducting a thorough examination of the patient
0.002	14.83	0	4	7	17	20	Changing and reforming the laws and regulations governing the country's health system
0.001	19.83	0	4	5	17	22	Establishing think tanks and presenting guidelines

## Discussion

This study was the first comprehensive investigation on the economic and clinical dimensions of routine tonsillectomy specimens for histopathological examinations in Iran. It was carried out on a large sample size over 15 years with the aim of investigating the necessity or unnecessity of sending all tonsillectomy specimens for pathology tests, the rates of malignancies, cost savings, and the reasons for sending all specimens to pathology laboratories, and the strategies for its prevention. 

From a clinical point of view, the results of the present study indicated that it was unnecessary to send all tonsillectomy specimens for histopathological examinations. In this regard, this result was consistent with the findings of the studies conducted by Ramzi et al., Faramarzi et al., Taner et al., Nester et al., Prim et al., Randall et al., Erdag et al., Verma et al., Dohar et al., Adoga et al., and Rebechi et al. in other countries on the dispensability of pathological examination of all specimens (2,3,6,10-16). 

Therefore, as the results indicated, among all the 18,437 cases examined during 15 years, only 118 (0.64%) abnormalities were diagnosed; moreover, 0.36% of the specimens had malignancies, and only 0.02% were <18 years old. In this regard, this result was in line with the findings of studies conducted by Faramarzi et al., Randall et al., Avaliako et al., Felix et al., Ardag et al., and Dohar et al. 

(9-13,16,17), who suggested that malignancies in tonsillectomy histopathology tests were sporadic and only abnormal and suspected cases needed to be sent to laboratories. The results also showed that physicians identified all abnormal cases, especially those with malignant masses, as suspected cases before sending them for pathological examinations due to clinical indicators. 

Accordingly, the evidence demonstrated that it was unnecessary to send routine specimens of all ages, especially children. Moreover, if there were other clinical findings of malignancies, the specimens would necessarily be sent to pathology laboratories to be examined.

This finding is consistent with the results of a study by Adoga et al., who concluded that there was a significantly low chance of malignancy in children and patients without risk factors (12). From an economic point of view, the results of this study showed that the mean direct costs of performing a histopathological examination were about $72 and $147 (PPP) in the public and private sectors, respectively, and the total costs of all histopathological examinations performed each year in the studied centers were $88,488 to $180,692 PPP. In addition, workforce expenditures accounted for the largest share of costs in both public and private sectors (63% and 73%, respectively). In this regard, the findings of the present study are consistent with the results of most studies on the costs in the health sector (18-20). From an economic dimension, few studies have been conducted on the pathology of tonsillectomy specimens. In a study conducted by Prim et al., the mean cost of each tonsillectomy pathology test was estimated to be $30 (15), and Schrock et al. estimated a $69 cost per specimen (4). In addition, as reported in an investigation carried out by Hobbs et al., each specimen's cost was estimated to be $60.67 (5); however, Rebechi et al. reported this cost to be $20.03 (14). As demonstrated, the cost of examining each specimen could be different depending on the economic conditions of countries.

According to the results, not sending routine cases for pathological examinations could save approximately $87,919-$179,530 of the annual financial resources for examinations only in the two public medical centers under study. In this regard, the results of the present study are consistent with the findings of most published studies, especially Prim et al., Younis et al., and Netser et al. (3,6,15), Therefore, on average, it could be mentioned that the diagnosis of any abnormal case among routine specimens cost $11,250-$22,972 PPP; however, if only suspected and nonroutine cases were examined, $72-$147 would be imposed on the health system. Accordingly, the cost of routine cases was higher than 156 times as much as that of nonroutine ones; therefore, the results indicated that examining only nonroutine cases was pathologically cost-effective, while it was not cost-effective to perform pathology in many common cases with appropriate clinical records. Therefore, the results of the present study were in line with the findings of the investigations carried out by Younis et al. and Netser et al. on the cost-effectiveness and cost-benefit of tonsillectomy histopathology, in which it was reported that sending all tonsillectomy specimens to laboratories was not cost-effective (3,6). 

The cost-saving on pathology reports for any tonsillectomy specimen in the country could be potentially significant; therefore, sending all specimens for histopathology would be costly considering the current situation.

Moreover, it is needed to consider other issues caused by the current situation, including the issues related to specialists and human resources, as well as the time spent. It is noteworthy that insurance companies reimburse a significant part of these financial resources, and due to the low economic status of insurance companies, prevention of the examination of all specimens can significantly help insurances and cause a redistribution of resources to patients' services.

Furthermore, the second part of the investigation demonstrated that with a 99% confidence interval, the ENT experts believed that the rules and regulations governing the country's health system and the suspicion of the presence of tumors were among the reasons for sending tonsillectomy specimens for pathology. However, doubts in the diagnosis of uncommon pathogens, patient insistence, pathologist's approval to complete the final process, and a routine procedure (normality) in the country are not the reasons for sending tonsillectomy specimens for pathology. 

This finding indicates that the issue needs to be resolved by reviewing the governing rules and regulations, and the role of suspicion of the presence of tumors must be considered. In addition, among literature, there is no study on this issue to compare the results with those of the present research.

With respect to prevention strategies, the participants suggested that taking complete records of patients, conducting thorough examinations on patients, modifying the rules and regulations governing the country's health system, establishing think tanks, and providing guidelines could help prevent sending tonsillectomy specimens for pathological examinations. It means that experts could prevent the events to some extent by considering these strategies.

The lack of access to the statistics on the number of tonsillectomies performed in one year in the public and private sectors throughout the country and the ENT specialists' incomplete responses to the questionnaire were the limitations of the present study. The total financial burden of sending routine tonsillectomy specimens would be estimated if the information were available. 

In order to eliminate this limitation, the statistics of tonsillectomies in 2018 in the public sector across Fars province, Iran, as one of the country's significant provinces, were obtained. Moreover, another limitation was the manual record of the reports in pathology notebooks, which increased the errors, which were reduced by investigating all suspected cases in the system.

## Conclusion

According to the economic-clinical results of this study, it could be concluded that for economic reasons, sending tonsillectomy specimens should only be limited to nonroutine cases. Moreover, sending routine specimens is not a cost-effective solution considering the ability of physicians to identify suspected cases and clinical findings indicating disease malignancy before sending them. Furthermore, according to the results of the second part, the rules and regulations governing the country's health system and the suspicion of the presence of tumors are among the reasons for sending tonsillectomy specimens for pathology. In addition, taking complete records of patients, performing thorough examinations on patients, modifying the rules and regulations of the country's health system, establishing think tanks, and providing guidelines could help prevent sending tonsillectomy specimens for pathological examinations. 
